# Autoimmune haematological disorders in two Italian children with Kabuki Syndrome

**DOI:** 10.1186/1824-7288-40-10

**Published:** 2014-01-25

**Authors:** Paola Giordano, Giuseppe Lassandro, Maria Sangerardi, Maria Felicia Faienza, Federica Valente, Baldassarre Martire

**Affiliations:** 1 University of Bari “Aldo Moro”, Department of Biomedical Sciences and Human Oncology, Paediatric Hospital, Piazza Giulio Cesare, 11 – 70124 Bari, Italy

**Keywords:** Kabuki syndrome, Immune haemolytic anaemia, Immune thrombocytopenia, Autoimmune haematological disorders, *KMT2D*, *MLL2*, *KDM6A*

## Abstract

Kabuki syndrome (also called Niikawa-Kuroki syndrome) is a rare genetic disease described for the first time in Japan, characterised by anomalies in multiple organ systems and often associated with autoimmune disorders and impaired immune response. We herein report the clinical history, the therapeutic approach and the outcome of two children with Kabuki syndrome who developed autoimmune haematological disorders (haemolytic anaemia and immune thrombocytopenia). Factors regarding differential diagnosis and interventions in better management of this syndrome and its complications are discussed. This is the first report of Italian children with autoimmune haematological disorders complicating Kabuki syndrome.

## Background

The condition named Kabuki make-up syndrome (KS) is a rare multiple anomaly syndrome. The name was attributed because of the facial resemblance to the make-up used in traditional Japanese Kabuki theatre. It was first described in 1981 by Niikawa [1] and Kuroki [2,3]. The estimated prevalence of KS in Japan was 1/32,000 people with almost equal sex distribution. Although it was initially considered as a disease affecting exclusively the Japanese population, several reports support a widespread ethnic distribution of KS [4]. The main clinical characteristics are distinctive facial features: elongated palpebral fissures with eversion of the lateral third of the lower eyelid; arched and broad eyebrows; short columella with depressed nasal tip; large, prominent, or cupped ears. Other findings may include: minor skeletal anomalies, persistence of fetal fingertip pads, from mild to moderate intellectual disability, postnatal growth deficiency, congenital heart defects, genitourinary anomalies, cleft lip and/or palate, gastrointestinal anomalies. Functional differences can include:seizures, endocrinologic abnormalities (premature thelarche in females, feeding problems, hearing loss), hypogammaglobulinemia, increased susceptibility to infections and autoimmune disorders as idiopathic thrombocytopenic purpura (ITP), haemolytic anemia, thyroiditis and vitiligo [5,6]. The prevalence of major findings is summarised in the table (Table 1) [7-12]. Genetic basis of KS has been recently elucidated. Mutations in the mixed lineage leukaemia 2 gene, *KMT2D* (formerly *MLL2*) mapping on chromosome 12q13.12, are described in 55-80% of KS patients [13]. KMT2D is a histone H3 lysine 4 (H3K4)-specific methyl transferase that belongs to the SET1 family of human SET-domain protein methyltransferase superfamily. KMT2D acts as a part of multiprotein complex named ASCOM. It interacts with oestrogen receptor-α and it is important for epigenetic transcriptional activation and for embryonic development [14]. KMT2D has emerged as one of the most frequently mutated genes in a variety of cancers, including lymphoma, medulloblastoma and gastric cancer, thus supporting a role as a tumor suppressor in various tissues [15]. In patients without *KMT2D* mutation, *KDM6A* gene mutations have been reported. Lederer and Miyake described, respectively, deletion and point mutations as cause of KS [16,17]. *KDM6A* located at Xp11.3 encodes the lysine demethylase 6A demethylating di-and trimethyl-lysine 27 on histone H3. Like KMT2D, KDM6A plays an important role in embryogenesis and development [18]. In a recent multicenter study short stature and postnatal growth retardation were observed in all individuals with *KDM6A* mutations, but in only half of the group with *KMT2D* mutations [19]. As KS is a multisystemic disorder, people with KS may require various diagnostic and screening tests, assessments, referrals and multidisciplinary interventions at different stages of their lives [9-12]. For these reasons in 2011, the panel of DYSCERNE team (a network of centres of expertise for dysmorphology), funded by the European Commission Public Health Executive Agency, wrote the “Kabuki Syndrome Clinical Management Guidelines” [20]. These guidelines have been developed using a robust methodology based on the one used by the Scottish Intercollegiate Guidelines Network (SIGN). The method has been adapted to suit rare conditions where the evidence base is limited, and where expert consensus plays a greater role. Of note in the natural history of the KS, an abnormal immune regulation may occur. Some authors reported their experiences in the treatment of KS patients with immunodeficiencies [21] or autoimmune disorders [22]. In this paper, we contribute to the list describing two Italian KS children with autoimmune haematological disorders and their clinical management.

**Table 1:** The prevalence of major findings in more than 350 individuals with Kabuki syndrome (KS)

**Finding**	**Prevalence (%)**
Typical facial features*	~ 95
Intellectual disability	92
Hypotonia	25-89
Postnatal growth retardation	35-81
Joint hypermobility	50-75
Feeding difficulties	70
Congenital heart defects	40-50
Premature thelarche	7-50
Hearing loss	40
Seizures	10-39
Ocular anomalies	33
Cleft lip and/or palate	33
Renal and urinary tract anomalies	25
Immune dysfunction:	~ 20
Hypogammaglobulinemia,	
Idiopathic Thrombocytopenic Purpura (ITP), Autoimmune haemolytic anemia,	
Thyroiditis	
Vitiligo	

*Elongated palpebral fissures with eversion of the lateral third of the lower eyelid; Arched and broad eyebrows; Short columella with depressed nasal tip; and large, Prominent, or cupped ears.

## Case 1

This female patient was diagnosed as KS when she was ten years old through genetic counselling. Her typical facial features were: large and low set ears, elongated palpebral fissures with eversion of the lateral third of the lower eyelid. Additional phenotypic malformations were: brachydactyly V, prominent fingertip pads, muscle hypotonia, joint hyperlaxity, gastroesophageal reflux, left convex scoliosis, moderate mitral stenosis. She was born preterm at 34^th^ weeks of gestational age by caesarean section. In the perinatal period she presented halting weight gain. At the age of three months it was detected a ventricular septal defect (VSD) and a mild aortic coartation, surgically corrected. During her first year of life,a delay of psychomotor development arose. She faced a new heart surgery when she was three years old for aortic decoartation. Screening of *KMT2D* gene (by PCR amplification of all the 54 exons spanning the *KMT2D* gene and then by high-throughput sequencing confirming with direct sequencing) [23] didn’t find any mutations, otherwise mutation analysis of *KDM6A* gene has not yet been performed. She was referred to our paediatric haematology unit at the age of eleven for relapsing of chronic immune thrombocytopenia, onset two years earlier and treated with courses of intravenous immunoglobulin and steroid. At this time laboratory investigations revealed: immune haemolytic anaemia (Hb 8.7 g/dl, direct and indirect Coombs test positive), thrombocytopenia (platelet count 4000/mm [3]) and partial defect of serum IgA levels (26 mg/dl). She received intravenous immunoglobulin at the dose of 0.8 g/Kg for two days in association with metil-prednisolone at the dose 10 mg/Kg i.v for three days. After tapering steroid therapy, she was discharged with partial haematological remission, (Hb 12.8 g/dl, Coombs test positive, Plt 13600/mm [3]) and started at home treatment with low dose of prednisone (2 mg/Kg/day). In the following years the patient experienced relapsing course of the autoimmune cytopenia which needed to change immunosuppressive therapy with cyclosporine achieving a sustained haematological remission. Nowadays the patient has a resolution of her thrombocytopenia but remains Coombs positive.

## Case 2

**Figure 1:**
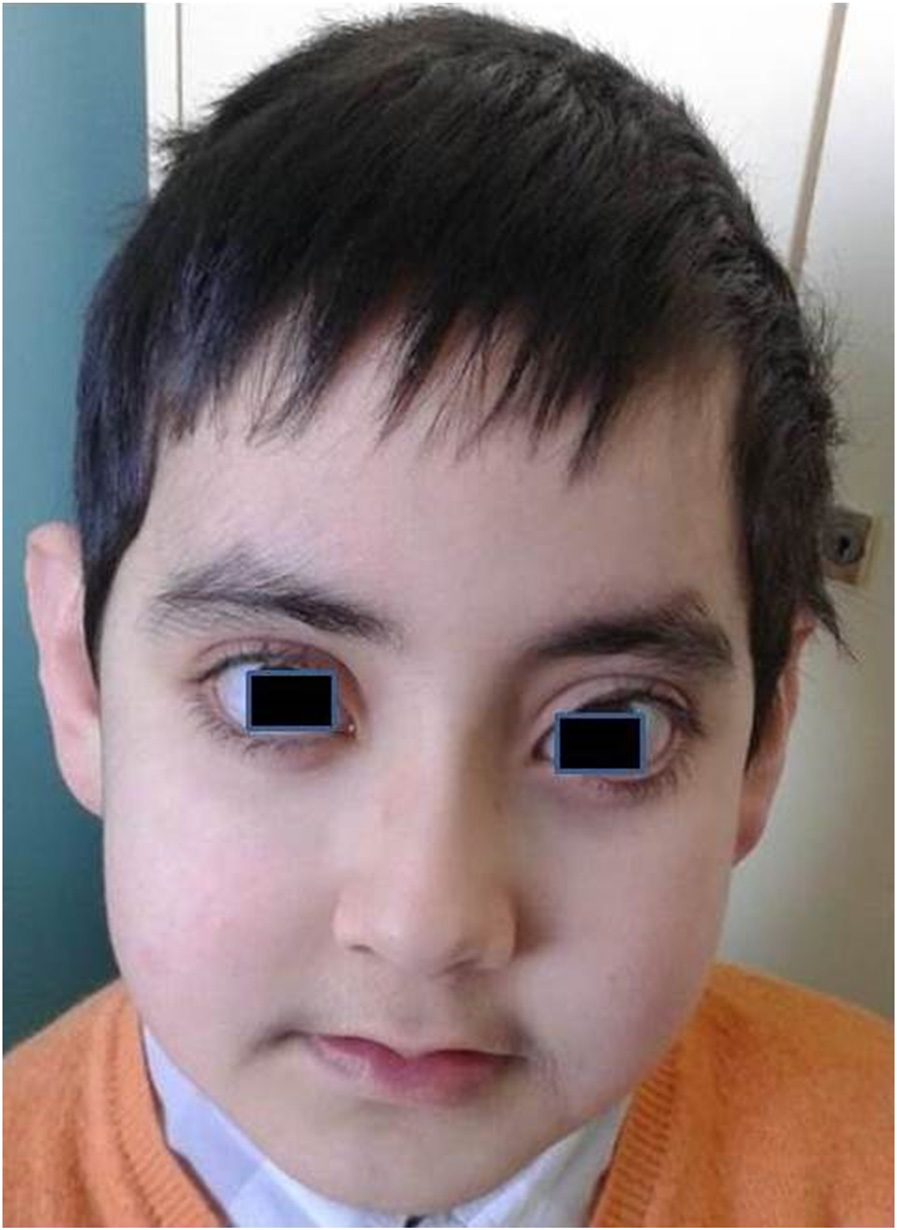
Typical facial features (front shot).

**Figure 2:**
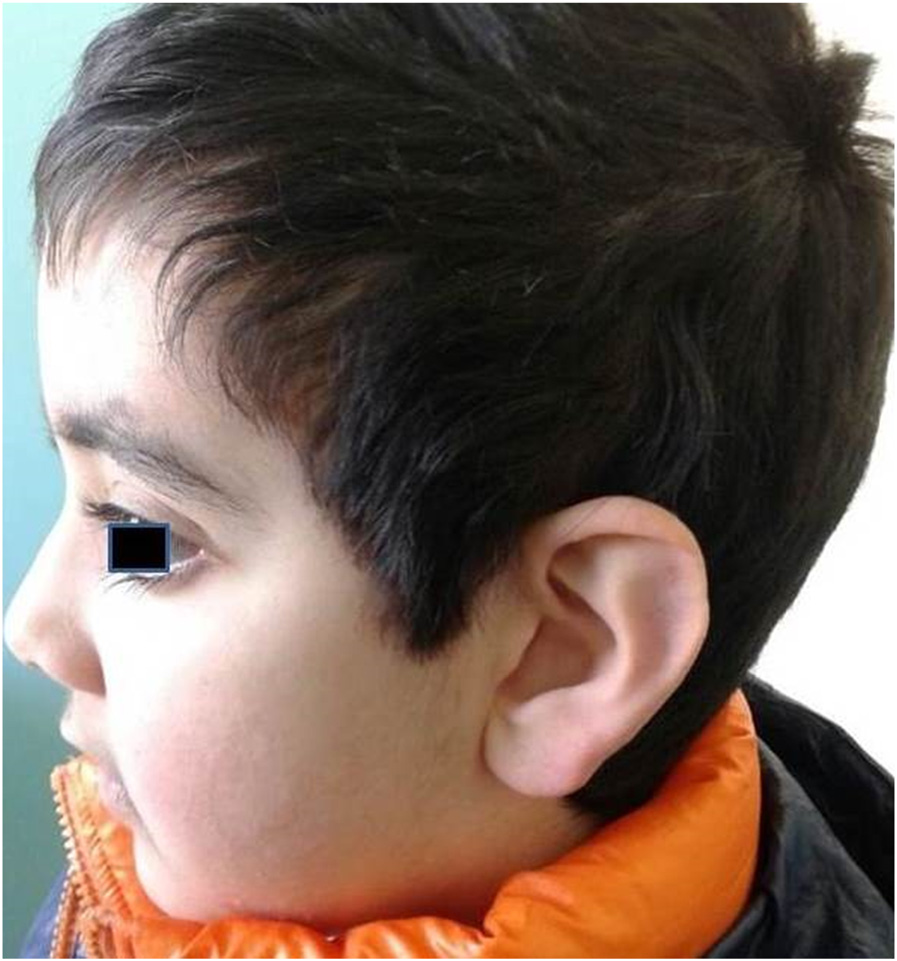
Typical facial features (profile shot).

This male patient presented hypodontia, lower lip pits, long palpebral fissures, prominent eyelashes, thinning of the central part of the eyebrow, mildly protuberant ears, prominent fingertip pads and hypospadias (Figures 1 and 2). He underwent surgery to correct palatoschisis and ventricular septal defect, at eight and eighteen months of age, respectively. At the age of nine he was admitted to our paediatric haematology unit for leukopenia and thrombocytopenia: his platelet count was 16000/mm^3^, and white blood cell count 1400/mm^3^. Serology for viral and microbiological infections was negative. Laboratory investigations revealed normal immunoglobulin levels and positive direct Coombs test. Bone marrow needle aspirate showed normocellular marrow and an increased number of immature megakariocytes, consistent with the diagnosis of ITP. Fluorescence in situ hybridization assay on bone marrow sample displayed no cytogenetic abnormalities in 7, 8, 21 chromosomes. To evaluate malformation framework, a high-throughput sequencing of *KMT2D* gene [23] was performed. The analysis showed a c.16384 G > C heterozygosis sequence variation resulting in the change of aspartic acid with histidine and causing the D5462H mutation. During hospitalisation, the patient received a single intravenous immunoglobulin administration, at the dose of 0.8 g/Kg, achieving a partial response of platelet count (98000/mm^3^). He was discharged with steroid home therapy (oral prednisone, 2 mg/Kg/day and progressive dose reduction in one month). During follow-up, he had a chronic and relapsing course of the autoimmune cytopenia, reason for which the study of lymphocyte apoptosis was carried out. Lymphocyte survival after Fas stimulation resulted normal and also the ratio of double negative (DN) [CD3+ TCRαβ + CD4-CD8-] T cells was within normal values (1%), thus ruling out an autoimmune lymphoproliferative syndrome (ALPS). Currently, three years after the first observation, the patient still has persistent leukopenia and relapsing episodes of thrombocytopenia, treated with recurrent courses of intravenous immunoglobulin and steroid.

## Discussion

KS is a multiple anomaly/mental retardation syndrome. In 2010, *KMT2D* was identified as the first causative gene of KS. On the basis of published reports, 55-80% of KS cases can be explained by mutations in these gene. Recently, also *KDM6A* deletions and point mutations have been detected in KS patients, however in 20 - 45% of cases the genetic basis remains unknown. Therefore different authors emphasises the search of additional KS causative genes [24]. This syndrome is sometimes associated with autoimmune abnormalities, such as idiopathic thrombocytopenic purpura (ITP) (from 1.6 to 17% of cases) [9], autoimmune haemolytic anaemia, leukoplakia, vitiligo and thyroiditis. Since the autoimmune disease does not occur until later childhood, and many of KS reports regard the paediatric age, the real frequency of autoimmune conditions in this syndrome might be underestimated. Moreover, KS patients have increased susceptibility to infection due to immune defects, particularly hypogammaglobulinemia [21]. Of note, the combination of immune deficiency and increased incidence of autoimmune disease has also been observed in patients with DiGeorge Syndrome (DGS) and Common Variable Immune Deficiency (CVID) [25-33]. The DGS is most commonly associated with chromosome 22q11.2 deletion and includes cardiac malformations and facial abnormalities, as well as KS, but also hypoparathyroidsm and T-cell deficiency [34]. On the other hand, the pattern of antibody abnormalities seen in children with KS resembles Common Variable Immune Deficiency (CVID). CVID is an heterogeneous immune disorder characterised by failure to properly induce B cell development and an increased incidence of autoimmune disease. Haematological disorders complicating KS have already been described [10,35], but to our knowledge, our cases are the first occurring in paediatric age in Italy. Diagnosis of Kabuki was made on the basis of facial features, confirmed in one case by mutation analysis of *KMT2D* gene. Since the high prevalence of autoimmune lymphoproliferative syndrome in children with idiopathic autoimmune cytopenias, [36], one out of two patients who had a partial defect of IgA, underwent the study of FAS-mediated apoptosis and the detection of DN lymphocytes. ALPS is a genetic disorder of FAS apoptotic pathway causing autoimmune cytopenia, organomegaly, lymphadenopathy and increased risk of malignancy. Interestingly, the autoimmune cytopenia associated with KS is often chronic. The conventional therapy (intravenous immunoglobulins and steroids) of this condition is often marked by lack of response and relapse of autoimmunity and/or therapy side effects. In particular, Torii et al. [35] showed the successful treatment with Rituximab of refractory idiopathic thrombocytopenic purpura. We treated our cases according the recommendations of Paediatric Oncology and Haematology Italian Association [37] keeping in mind the risk for these patients to develop hypogammaglobulinema [21]. The use of different immunosuppressive protocols, chosen on the experience of each single centre, should lead medical community to standardise therapeutic approaches for KS. Collecting data, concerning a considerable number of patients, about diagnosis, clinical, therapy and outcome might help to improve the management of KS patients as the French registry demonstrates [38]. In addition, due to the underlying immune dysregulation, we suggest to perform a immunologic evaluation at the time of diagnosis, in order to reduce preventable morbidity in these patients.

## Consent

Written informed consent was obtained from the parents of patients for describe these cases report and to publish their pictures. A copy of the written consent is available for review by the Editor-in-Chief of this journal.

## Abbreviations

KS: Kabuki make-up syndrome; VSD: Ventricular sept defect; ITP: Idiopathic thrombocytopenic purpura; CVID: Common variable immune deficiency; DN: Double negative [CD3+ TCRαβ + CD4-CD8-] T cells; ALPS: Autoimmune lymphoproliferative syndrome

## Competing interests

The authors declare that they have no competing interests.

## Authors’ contributions

MB and LG wrote the draft version. All authors read, revised and approved the manuscript.
